# El paso de la pandemia en un hospital: covid-19 y muerte en Buenos Aires, Argentina

**DOI:** 10.1590/S0104-59702023000100048

**Published:** 2023-09-18

**Authors:** Sandra Gayol, Maximiliano Ricardo Fiqueprón

**Affiliations:** i Consejo Nacional de Investigaciones Científicas y Técnicas Buenos Aires Argentina sandra.gayol@gmail.com Investigadora principal, Consejo Nacional de Investigaciones Científicas y Técnicas . Buenos Aires – Argentina sandra.gayol@gmail.com; ii Consejo Nacional de Investigaciones Científicas y Técnicas Buenos Aires Argentina fiquepronmaximiliano@gmail.com Investigador asistente, Consejo Nacional de Investigaciones Científicas y Técnicas . Buenos Aires – Argentina fiquepronmaximiliano@gmail.com

**Keywords:** Covid-19, Espacio hospitalario, Practicas profesionales, Muerte, Covid-19, Espacio hospitalario, Practicas profesionales, Muerte

## Abstract

El artículo explora las mutaciones en las prácticas de los profesionales de la salud en el contexto de la covid-19. Se centra en “el área covid” de un hospital de la provincia de Buenos Aires, Argentina, buscando conocer el reordenamiento del espacio y rutinas hospitalarias, y las formas de comunicar la muerte. En un período corto de tempo se observan ajustes en las prácticas profesionales e intentos de nuevas rutinas y rituales. En el vínculo médico/paciente y en la forma de comunicar una muerte se condensan tanto las acciones “excepcionales” (que vulneram rutinas) como intentos de recuperar técnicas de cuidado vinculados al paradigma de la medicina humanizada.

## Abstract (en)

The article explores the mutations in the practices of health professionals in the context of covid-19. It focuses on “the covid area” of a hospital in the province of Buenos Aires, Argentina, seeking to know the rearrangement of hospital space and routines, and the ways of communicating death. In a short period of time, adjustments in professional practices and attempts at new routines and rituals are observed. In the doctor/patient relationship and in the way of communicating a death, both “exceptional” actions (which violate routines) and attempts to recover care techniques linked to the paradigm of humanized medicine are condensed.

En el 2015, Margaret Chan, directora general de la Organización Mundial de la Salud (OMS), afirmaba que más allá de los interminables desafíos sanitarios, una tendencia mundial era indiscutida: el envejecimiento de la población se está acelerando en todo el mundo. Por primera vez en la historia – afirmaba –, la mayoría de las personas pueden aspirar a vivir más allá de los sesenta años (OMS, 2015). El impacto de este proceso se plasmaba en otra novedad: un número cada vez mayor de seres humanos mueren hoy de enfermedades degenerativas de la vejez, de afecciones cardíacas y/o de diferentes tipos de cáncer. Estas dolencias, que en general transitan durante años y meses, desplazaron a las enfermedades infectocontagiosas y dejaron de tener como escenario la habitación del enfermo en su hogar (como era habitual en el siglo XIX) para trasladarse a los hospitales. Aquí, las salas de guardia, las de internaciones y los quirófanos (de la mano de saberes, tecnologías y profesionales cada vez más especializados) conformaron una trama compleja de interacciones, prácticas y expectativas en torno a la vida y la muerte desde mediados del siglo XX. Los gobiernos, por su parte, también definieron sus prioridades y políticas de salud que marcaron la agenda pública a partir de estas mismas enfermedades ( Porter, 1997 ; Armus, 2007 ; Chiara, 2018 ).

El descenso de las tasas de mortalidad y las mejoras en la expectativa de vida en los años 1940 hicieron, por ejemplo, en Argentina y en otras regiones de Latinoamérica, que la muerte no sea vista, olida, oída y tocada por la población (Carbonetti, Celton, 2007). Parte de este escenario quizás hoy ya sea parte del pasado. Nuestra sociedad post-infecciosa vuelve a vivir con la covid-19 experiencias epidémicas similares a aquellas que ocurrieron durante el siglo XIX y XX. La muerte deviene omnipresente en nuestros pensamientos y acciones y se alteran los procesos de morir, de muerte y de duelo.

Junto a las cifras, gráficos y algoritmos que en tiempo real registraban la cantidad de infectados y muertos en las regiones más disímiles del planeta, numerosos trabajos se interesaron en los impactos que la nueva enfermedad covid-19 ha tenido y aún posee desde la perspectiva de la salud como un derecho humano, en los ritos mortuorios y la experiencia de duelo (Silva, Rodrigues, Aisengart, 2021; Irrazabal, Olmos, 2020; Pecheny, 2020 ). Surgieron estudios que indagaron, mediante encuestas, en una etapa muy temprana (primera semana de abril de 2020), cómo percibió el personal de salud del sistema público, privado y de seguridad social de la Argentina la preparación de sus instituciones para enfrentar la pandemia ( Ortiz et al., 2020 ). También se comenzó a explorar cómo los profesionales vivieron los cambios impuestos por la pandemia en un centro de atención primaria del conurbano bonaerense ( Freidin et al., 2021 ). Este artículo se inscribe en estas últimas preocupaciones y se pregunta, precisamente, cómo las rutinas diarias de tratamiento de la enfermedad, del proceso de morir y de comunicación de la muerte se alteran y al mismo tiempo buscan estabilizarse en un contexto de crisis que, por definición, es incierto y muy dinámico. Para ello nos focalizamos en “el área covid” de un hospital de la provincia de Buenos Aires, Argentina, buscando conocer el despliegue de preparativos y acciones que un conglomerado diverso de actores implementó entre marzo de 2020 y agosto de 2021. ^1^ Nos interesa poner el foco en “momentos” específicos: esperar la enfermedad, atender pacientes con covid-19, acompañar el proceso de morir y comunicar la muerte a las familias y/o cuidadores. Si todos los actores que trabajan en el hospital tienen información, saberes y roles sobre estos “momentos”, sus prácticas y acciones concretas estaban, y seguirán estando, claramente delimitadas por un campo profesional muy especializado y jerárquico.

Pensamos al hospital como un órgano de la sociedad que comparte sus características, que cambia a medida que la sociedad se va transformando. La enfermedad, evento biológico y social, refleja la historia intelectual e institucional de la medicina y puede ser una ocasión para legitimar políticas públicas, destacar aspectos sociales y sancionar valores culturales ( Rosen, 1985 ). Es un elemento decisivo y estructurante en las interacciones entre médicos y pacientes y puede generar nuevas instancias institucionales y prácticas profesionales que trasciendan la situación crítica que las estimuló. Por ello la pregunta por las permanencias y los cambios, la intensidad y la valoración que de los mismos tienen los actores directamente involucrados en el proceso de gestión de la crisis es central en nuestra indagación ( Cueto, 1997 ; Armus, 2002 ; Rosenberg, 1992 ).

En un período corto de tiempo – poco más de un año y medio – en donde la incertidumbre, el aprendizaje sobre algunas características de la enfermedad y sobre las formas más adecuadas de tratamiento estaban en permanente reconsideración; se observan no solo ajustes en las prácticas profesionales sino también intentos de rutinización y de ritualización. Esto último es evidente en la institucionalización de un esquema conversacional para una circunstancia imprevista y traumática como lo es la muerte por covid-19. ^2^ En este escenario doloroso tanto el médico como los familiares o cuidadores de los hospitalizados participan y aprovechan de los múltiples canales de la interacción social. Es en la forma de comunicar una muerte, profundamente alterada por la situación crítica de la pandemia, donde se percibe tanto el margen para las acciones “excepcionales” que vulneran las rutinas como intentos de recuperar técnicas de cuidado vinculados al paradigma de la medicina humanizada. La “cuota personal de cada uno” y las creencias individuales también se sumaron como recursos para quienes gestionaron el tratamiento de la nueva enfermedad y formas de morir.

El artículo se organiza en tres apartados, precedidos por una breve aclaración metodológica, y una conclusión. En el primer apartado analizamos la reconfiguración del espacio del hospital estimulado por la crisis; en el segundo ponemos el foco en las mutaciones de las prácticas profesionales para atender a pacientes con covid-19; y en el tercer apartado nos detenemos en las interacciones de los médicos y médicas con los allegados del paciente y en las maneras de comunicar la muerte. En la conclusión retomamos algunas ideas generales y la autoevaluación que los actores hicieron sobre su performance en la gestión de la pandemia.

## Fuentes y metodología

Las principales fuentes impresas consultadas son los protocolos y normativas dictadas por el Ministerio de Salud de la Nación Argentina y por la OMS, las publicaciones del Hospital Nacional Profesor Alejandro Posadas (Hospital Posadas) y los periódicos de cobertura nacional disponibles *on-line* . ^3^ Una fuente muy importante de información proviene de la observación de los Encuentros Hospitalarios de Gestión ^4^ y de las 23 entrevistas al personal de salud que trabajó en el “área covid”: médicos y médicas de guardia respiratoria, clínica médica, unidad coronaria y unidades de cuidados intensivos; enfermeras y enfermeros, profesionales integrantes del equipo de conducción y gestión del hospital, camilleros y choferes de ambulancia afectados al trasladar pacientes con covid-19. El cuestionario base para iniciar las entrevistas fue aprobado por el comité de ética del hospital. La guía de preguntas semiestructuradas fue readaptada parcialmente en función de los perfiles profesionales y los roles en la institución de las personas entrevistadas. Se contempló la perspectiva de género, el tiempo de ejercicio en la profesión y en el Hospital Posadas, la especialidad y el lugar de cada uno en la escala jerárquica. ^5^ Las y los entrevistados, entre mayo y agosto de 2021, sabían que la conversación giraría en torno a su práctica profesional antes y durante la pandemia y a su relación con la muerte. ^6^ Estos ejes temáticos disparadores del intercambio inicial fueron profundizados con momentos significativos para los protagonistas: su primer contacto con un paciente positivo de coronavirus, el aprendizaje en el uso del traje de protección y en torno al nuevo virus, el contagio, el primer paciente muerto y experiencias con la muerte. En cada uno de ellos se buscó captar una diversidad de perspectivas ya que la experiencia nunca es unívoca y, al mismo tiempo, se buscó ponerlas en relación con un contexto mayor.

Se trata de un estudio de caso cualitativo que, como todos los estudios de caso, permite abordar de manera situada un fenómeno complejo y que atento al contexto puede dar cuenta de relaciones y procesos en escenarios sociales particulares ( Yin, 2014 ; Stake, 1995 ).

## El espacio hospitalario en tiempos de crisis

La “adaptación del espacio físico”, en palabras del entrevistado P., es parte de los cambios que suceden durante períodos epidémicos. Sabemos bien que, durante el siglo XIX, por ejemplo, fueron usuales las construcciones de lazaretos, pabellones y secciones para tratar a los enfermos de una dolencia específica ( Álvarez, 2004 ; Santos, 2007 ). Entrado el siglo XX, los pabellones para tuberculosos y para enfermos de poliomielitis también destinaron áreas para saberes expertos, definiendo una cultura espacial de la salud que encuentra en los hospitales un lugar privilegiado para representar la lucha contra estos males (Ramacciotti, Testa, 2021; Testa, 2018 ; Rogers, 2013 , Oshinsky, 2005 ).

El Hospital Nacional Profesor Alejandro Posadas se pensó, precisamente, en este contexto. Inaugurado en 1958, aunque la iniciativa fue del gobierno de Juan Perón, su diseño arquitectónico original con espacios abiertos y balcones orientados para permitir el reposo del paciente frente al sol (todavía en funciones), se debió a que fue imaginado para atender afecciones pulmonares y respiratorias, especialmente por tuberculosis. Sin embargo, combinó desde sus inicios la atención de diversas patologías con la investigación. Este hecho lo convirtió en un referente nacional para la atención de enfermos agudos en todas las etapas de la vida. Actualmente trabajan más de cinco mil personas y su principal población hospitalaria proviene de quienes habitan en los municipios que lo circundan y que albergan casi seis millones de habitantes. ^7^

**Figura 1:**
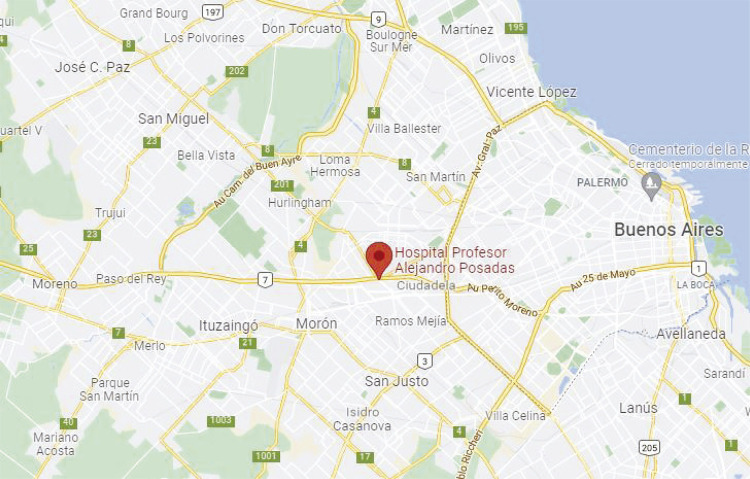
: mapa delimitando zonas de influencia del Hospital Posadas (Fuente: Google Maps. Disponible en: https://www.google.com.ar/maps/@-34.6315323,-58.5746395,13z?entry=ttu. Acceso en: 12 jun. 2023.)

Este “mamotreto” e “ícono”, como lo definió una entrevistada, fue rediseñado cuando la OMS decretó la pandemia generada por el virus SARS-CoV-2. La transformación, producto de la necesidad de enfrentar la crisis, produjo dos hospitales en un hospital. Una puerta de ingreso, la “de siempre y normal” (entrevistada L.), ^8^ y aquella habilitada para los sospechosos de estar infectados con el nuevo virus. La guardia respiratoria es, en efecto, la vía de ingreso de las personas que tienen algún síntoma compatible con la covid-19. Distante de la puerta principal de acceso al “mamotreto”, ventanas y vidrios separan a su personal de potenciales internados y ofician de prólogo de la segregación espacial reinante en las salas con pacientes. Estas salas fueron, a su vez, reagrupadas. Una parte de clínica médica fue exclusivamente destinada a pacientes con covid-19 y las terapias intermedias y las intensivas fueron unidas. En abril de 2020 se inauguró una nueva sala de terapia con setenta camas. ^9^

Las experiencias institucionales previas fueron reactivadas para este rediseño espacial. Especialmente la vinculada con la gripe H1N1 del 2009 durante la cual el nosocomio y sus profesionales, muchos de ellos todavía en funciones, lideraron la gestión de la crisis a nivel nacional. Así, se creó el área o zona “covid” y el área o zona “no covid” que separa y determina qué es peligroso e incide en las percepciones de los riesgos.

– En general cuando hay una pandemia de este tipo los otros especialistas huyen. A nadie le gusta acercarse. Es más, el año pasado [2020] si un intensivista caminaba por el pasillo los demás médicos se le alejaban.

– ¿Por qué?

– Por factor de contagio. Ahora este año no [2021], pero el año pasado nuestros propios colegas se alejaban, se alejaban, ahora eso no porque están todos vacunados… [ *sic* ] es más, a mí había lugares que no me dejaban ingresar, me decían bueno no entres vos atendes enfermos, me podés contagiar [se ríe] era parte del principio de la pandemia.

– ¿Y vos qué decías?

– Y uno se siente mal… [ *sic* ] después eso pasó, pero bueno, es parte del miedo (entrevistada C.A.). ^10^

Las percepciones de los riegos no son estables en el curso del tiempo, tampoco unívocas, y como se desprende del testimonio de C.A. citado arriba, que sitúa el hecho que narra en la entrevista “entre marzo-abril del año pasado más o menos [2020]”, no obedecen siempre a una evaluación racional por parte de quienes las realizan ( Douglas, 1992 ).

En efecto, el “área covid” es un espacio físico, material, que porta también su condición de frontera entre lo impuro – su interior – de lo puro e incontaminado – el afuera. Las formas de protegerse de lo “sucio” como muestra el testimonio de C.A., y los dos testimonios que le continúan, generaron evaluaciones que se asentaron en criterios científicos, pero también en creencias que circulaban en el espacio público. Las formas de sociabilidad previa, entonces, se trastocan. En primer lugar, porque ambas zonas no se comunican entre sí. En segundo lugar, porque “los no covid”, como cuenta C.A., evitaban contactar a los “covid”. Las percepciones de los riesgos por parte de una población heterogénea, y como han demostrado numerosos trabajos, son muy diversas. No se sustentan solo en saberes biomédicos ni en una racionalidad orientada por la probabilidad estadística de que un determinado evento futuro sobrevenga, por ejemplo, el peligro individual a contagiarse o a morirse (Douglas, Wildavsky, 1982). Ante una nueva enfermedad y cuya forma de transmisión todavía hoy es objeto de discusión incluso científica, la demarcación calificaba también entre el personal hospitalario que “atiende enfermos”, como cuenta C.A., y el restante personal del hospital que no se involucra con pacientes con covid-19. Este muro material, relacional y simbólico separó la pureza, lo limpio y lo sano; de lo impuro, sucio y enfermo (Douglas, Wildavsky, 1982).

con la covid-19 nosotros sentíamos que todo lo que estaba adentro de la sala estaba contaminado… no sé hasta qué punto eso es comprobable, pero en ese momento [se refiere a abril 2020] no había quien dijera lo contrario. … de hecho el hospital puso unos blindex para aislar ese sector limpio en el cual preparar la medicación. … Nosotros ahí nos sentíamos completamente seguros y mira que loco ¿no? porque yo lo veo a la distancia y la seguridad es entre comillas porque si el virus entraba ahí adentro estaba ahí adentro ¿de qué te protegía? pero bueno eran sensaciones que uno tenía ¿no? (entrevistada L.). ^11^

al entrar la primera vez, [a la sala con pacientes covid de clínica médica en abril de 2020] cada vez que venía el viento sentía que venía el covid; pero ese miedo fue pasando (entrevistada A.). ^12^

De esta manera el miedo, junto con la gestión del contagio, se transforma en otro ordenador del espacio, de las prácticas profesionales y los juicios personales. Los miedos que abruman la conciencia, entendidos como percepciones de daños, posibles resultados de las amenazas de inseguridad e incertidumbre de objetos, personas y situaciones, son parte de la experiencia humana y de las experiencias en pandemia ( Delumeau, 1978 , p.22). No se trata de un solo miedo, no es fijo, los miedos son dinámicos y dialogan además con un conglomerado de emociones (frustración, estrés, rabia, tristeza, ansiedad, impotencia, alegría) que tienen un lugar explícito en las narrativas individuales femeninas. En los varones la frase “sí tuve miedo” irrumpe cuando se les pregunta expresamente. No es un miedo por sí y por la muerte de si, “yo no tengo miedo a la muerte ni siquiera a la mía” es parte del discurso hospitalario (entrevistada M.E.); ^13^ se trata del miedo a contagiar a sus entornos próximos, a la familia y especialmente a los hijos (entrevistada A.P.). ^14^

– ¡Teníamos pánico!

– ¿De contagiarse?

– No tanto de contagiarnos nosotros, de llevar la enfermedad a casa porque nosotros estábamos todo el tiempo expuestos. En mi caso tengo a mis padres que son grandes… miedo a lo desconocido. No conocíamos a ese enemigo que estaba adelante nuestro y que hacía estragos … si bien uno veía que pasaba, no sabía cómo nos podía afectar a nosotros… uno siempre tiene la idea que le pasa al resto no a nosotros (entrevistada H.). ^15^

El miedo, y su versión más intensa y radical como el pánico y el terror, así como la impotencia, podía ser porque “nunca terminas de conocer al bicho” (entrevistada M.E.).

como digo, yo, el “saber” no te alcanza y sabes muy poquito, no tenés elementos científicos para actuar… se muere gente que en una situación normal no se hubiera muerto. Lloramos todos el 28 de diciembre [de 2020] cuando llegaron las vacunas al hospital (entrevistada M.E.).

La experiencia europea, sobre todo de España e Italia durante los meses de marzo y abril de 2020, prefiguró expectativas sobre las posibilidades del desarrollo de la covid-19 en Latinoamérica. Así nació la metáfora del “tsunami”, que se moduló en otras expresiones similares como “la pandemia que arrasa” o la “tragedia” que se nos aproxima.

ellos [se refiere a terapistas de Israel] están preparados a tratar con catástrofes. ‘¿Qué nos iba a pasar a nosotros’? (entrevistada C.A.).

– Teníamos terror. Vos veías eso…

– ¿Qué veías?

– Lo que estaba pasando, cuando veíamos lo que estaba pasando… [se agarra la cabeza]

– ¿Qué pasaba?

– Todo saturado. La gente se moría (entrevistada A.N.). ^16^

Todos los entrevistados convergen en colocar a las conductas del miedo – que los tuvo como sujetos y objetos – como parte de sus emociones, de sus valoraciones morales sobre el accionar de sus pares no afectados a la zona covid y como orientador de sus prácticas profesionales.

Como “fuerza estimulante” ( Robin, 2009 ) este miedo fue central para la reorganización, la toma de decisiones y el ejercicio del mando. También una alerta para intentar hacer lo correcto con la información disponible en ese momento. El Aislamiento Social Preventivo y Obligatorio (Aspo) – cuya evaluación retrospectiva es muy positiva – permitió “prepararnos, ir ganando confianza” y generar nuevas instancias organizativas para una gestión más eficiente y ordenada de la crisis. ^17^ En esta coyuntura específica se creó el Comité de Crisis o el “Comité covid” abocado, precisamente, al diseño y planificación de una “estrategia global covid-19”. Su primera prueba piloto fue en abril de 2020, y entre mayo y agosto de ese año – coincidente con la primera ola en el país – implementó diferentes procesos acordes con la evolución epidemiológica y la infraestructura disponible. Se inicia como un espacio pequeño integrado por los siete directores generales, luego se suman los jefes de servicio y “rápidamente arribaron todas las personas con capacidad técnica y de gestión para [luego] devenir un espacio abierto, interdisciplinario y con posibilidades de participación presencial o remota en sus reuniones periódicas” (entrevistadas M.E y C.A.). Si al comienzo los equipos compartían decisiones de acción, se sumó luego el monitoreo y las propuestas de mejoras y de reajustes estimulados por la propia dinámica de la situación pandémica:

Empezó siendo un espacio chiquito, destinado a covid-19 pero ahora [mayo de 2021] se presentan las diferentes estrategias que el hospital fue desarrollando para atender a la pandemia hasta ese momento, y después empezamos a generar espacios para que los equipos sean los protagonistas y contaran sus propias experiencias. Vamos seleccionando estratégicamente a distintos equipos del hospital con un mes de anticipación para que se preparen y hagan exposiciones (entrevistada M.C.A.). ^18^

Muy conocido entre los trabajadores del hospital, el Comité es un ejemplo de “institucionalización” que suelen propiciar las situaciones críticas ( Fiquepron, 2020 ) y una instancia institucional que, todos los testimonios convergen, se debe preservar en la “post-pandemia”.

## Rutinas laborales alteradas

Las redistribuciones, prolongaciones, umbrales y salidas que sintetizan la nueva gramática socioespacial se entrelazan con prácticas profesionales alteradas y modificaciones en el trabajo cotidiano. El concepto de “crisis” que, si bien puede ser parte de la práctica profesional de algunas especializaciones entre el personal de salud en “situaciones sociales normales”, con la covid-19 se impone por su carácter absoluto y su generalización. Comprende situaciones que suponen una discontinuidad del flujo temporal y un quiebre con el momento previo, visto como “normal” ( Koselleck, 1988 ). La particularidad de la crisis modelada por la pandemia es que las alteraciones radicales no sustraen al personal afectado al área covid del hospital de su rol clave en la gestión y solución de la crisis. Sus prácticas deben enmarcarse, especialmente el inicio, en una relación nueva con el saber experto. La formación continua y la especialización, inherentes al personal de salud, es desafiada por un virus nuevo cuyo saber sobre el comportamiento, diagnóstico y tratamiento eran muy escasos y estaban en permanente revisión ( Ramacciotti, 2021 ).

Del entrecruzamiento de las entrevistas con los protocolos y directivas del Ministerio de Salud de la Nación y las disposiciones de la propia institución, se advierte una multiplicidad de fuentes de información y formación que trascienden las instituciones oficiales y profesionalmente reconocidas. Como otrora, lo “que hay que hacer en el hospital” se transmite jerárquicamente desde las jefaturas hacia abajo.

Mientras avanzaban los contagios y las muertes en Asia y Europa y la OMS caracterizaba, el 11 de marzo de 2020, a la situación generada por la covid-19 como una pandemia, en el Posadas “valía todo”:

La información científica, los rumores, diarios, correlacionar bibliografía, averiguar cómo se puede estudiar (entrevistada M.E.).

Mirar videos subidos a las redes, seguir páginas de Instagram… hablar con colegas de España y de Italia… (entrevistada C.). ^19^

Esto está lejos… no se sabía bien qué era, leíamos informes, pensábamos que era una gripe como la del 2009 (entrevistada M.E.).

La experiencia práctica, “nuestra propia experiencia”, como me explica la entrevistada M.C., “hizo que fuéramos cambiando y no seguimos lo de Europa. Al principio había que intubar, después no, depende”. La flexibilidad en las decisiones tomadas en escenarios de trabajo en mutación constante fue una manera de gestionar la crisis y, retrospectivamente, es visto como un valor positivo tanto por personal médico como de enfermería. ^20^

Los aprendizajes suceden en tiempos convulsos, encarnados en cuerpos con nuevos ropajes cuyas formas de uso también fueron progresivamente adquiridas. El equipo protector compuesto por barbijo N95, gafas, barbijo genérico, máscara facial, cofia, camisolín emorepelente y guantes estériles, resignifica las prácticas y suspende transitoriamente conocimientos interiorizados, como las jerarquías. Es difícil distinguir para un ojo no avezado al jefe de clínica médica del residente o del enfermero. El uso de esta vestimenta no supone una novedad en sí misma, pero sí es nueva su recurrencia y obligatoriedad. Los brotes del ébola (el último en 2013), el SARS (2002-2003), la gripe aviar del 2005 y la gripe H1N1 de 2009, por ejemplo, proveen imágenes del pasado que se reactualizan y circulan globalmente. La visibilidad y masificación que estas imágenes logran producto de su circulación en redes sociales y medios de comunicación estabilizan un registro del peligro a la vez que reconstruyen una representación que lo asocia en sus cuidados casi exclusivamente con el médico. Son médicas y médicos, excepcionalmente enfermeros y personal de limpieza, los que estarían “detrás de los barbijos” y en la zona de mayor riesgo y gravedad.

Detrás de la vestimenta la escucha es difícil y también la conversación. Los entrevistados afirman que debieron suprimir “hablarles, preguntarles a los pacientes” (entrevistado P.) y ante la crisis priman “las evidencias a distancia” (entrevistada C.A.):

Es terrible. Sabemos que cuanto más tiempo de permanencia en la sala mayor posibilidad de contagio (entrevistada C.).

Nosotros estábamos muy acostumbrados a recorrer al lado del paciente, te cuenta por qué vino, los datos, agregamos preguntas, lo revisamos al lado del residente. Ahora se hacen todas las novedades en un aula. En un corto tiempo, pero efectivo hay que hacer preguntas y después pasamos al teléfono (entrevistada C.P.). ^21^

Este nuevo escenario para el diagnóstico incorpora nuevos dispositivos que reorganizan los contactos del médico con el enfermo y con sus familiares. “Pasamos horas hablando por teléfono y completamos planillas y formularios *on-line* ”, cuenta la entrevistada C., “hay doble o triple trabajo, se multiplicó el estrés. Siempre decimos ‘que fácil era esto antes’” (entrevistada C.A.).

La fugacidad del trato con el paciente, primer cambio que todos los entrevistados destacan como más relevante, tensiona el discurso y la práctica del personal de salud que de la mano del Movimiento Hospice introdujo también en la Argentina la noción de humanización de los cuidados. El eje conceptual del Movimiento Hospice, centrado en la dignidad de la persona ( Radosta, 2019 ), articulado con las propuestas de la medicina paliativa que aboga por el manejo de los síntomas y del dolor sobre la base de la comunicación médico-paciente, resultaban imposibles en pandemia ( Alonso, 2011 ). La vida digna se fue imponiendo en un discurso y lentamente en la práctica médica cada vez más distante, no obstante, del ensañamiento terapéutico y de la hegemonía del cuerpo médico en la toma de decisiones. ^22^ La pandemia en el espacio hospitalario imposibilita “de golpe”, sostiene J., la “humanización de los cuidados de la que veníamos hablando hace años” (entrevistado J.). ^23^

En la Unidad de Cuidados Intensivos, desde su creación a mediados del siglo XX, el equipo de salud se comunica poco con los pacientes (por lo general se encuentran sedados o con asistencia respiratoria), el contacto es con el familiar o el cuidador. Este vínculo se resquebrajó especialmente.

“Contacto físico ya no hay. Todo es telefónico. Hemos humanizado [junio de 2021] y cuando sabemos que un paciente va a fallecer lo hacemos venir al familiar”, nos comenta la entrevistada C.A. Repreguntamos entonces: ¿Quiénes son la familia? ¿Quieren venir al hospital? Los testimonios convergen “en general sí” y, al mismo tiempo, “depende”, “no todos”. Esta paleta de posibles parece relacionarse – además de con el vínculo posible entre el paciente y su entorno inmediato imposible de reconstruir – con la edad del familiar y con el momento de la pandemia. “La gente más grande, no quiere ir” especialmente por miedo al propio contagio o porque no siempre se valen por sus propios medios. ^24^ La vacunación, iniciada a principios de 2021, o la reconfiguración del vínculo que la propia persona mantiene con el virus alienta visitas a los enfermos e intentos de restituir mediante ellas el hecho afectivo. La presencia de la familia, cuidadores o allegados a un enfermo no es un universal y en pandemia, como relató la entrevistada H., “hubo casos en que todos los miembros de una familia estaban internados con el virus”.

## Comunicar la muerte

En el espacio hospitalario se aprende a ver la muerte cuando, en el curso de la experiencia con personas gravemente enfermas, el personal estudia y se entrena en detectar signos que signifiquen un orden particular de predicciones de la muerte. Los datos estadísticos acumulados también proveen un sistema de expectativas temporales en virtud de las cuales aquella puede preverse. Las enfermedades y los acontecimientos biológicos son agentes de pronóstico, de la cura, del proceso de morir y de la muerte. ( Sudnow, 2015 , p.59). Esta relación rutinaria, que no le quita a la muerte una textura de significados, también se trastoca en pandemia. El primer quiebre es, por supuesto, la recurrencia y el volumen de muertes. Según datos de la comisión epidemiológica del hospital, el promedio de muertes de los últimos años giraba en torno a los 1.100 fallecimientos por año. Entre abril del 2020 y mayo de 2021 se registraron 1.676 defunciones de las cuales 457 estuvieron vinculadas con la covid-19. ^25^ Estos valores acompañan la tendencia nacional que concentra la mayor cantidad de defunciones en los meses de julio a noviembre del 2020 (266 defunciones acumuladas por covid-19) y abril a mayo del 2021 (122 defunciones acumuladas).

**Figura 2:**
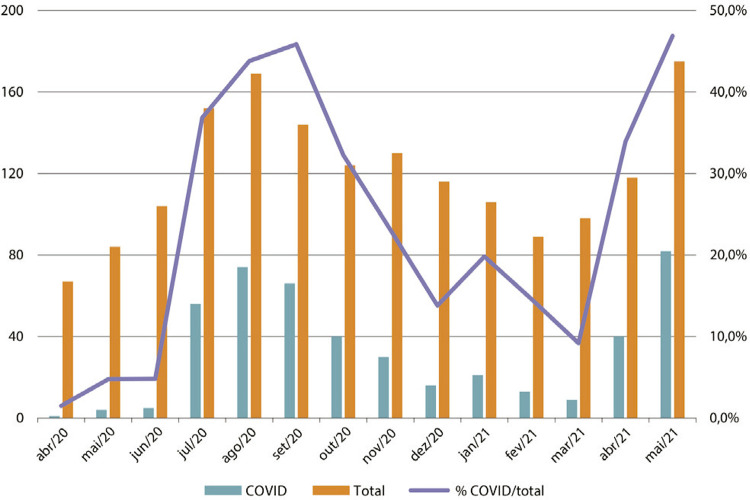
: Enfermedad por coronavirus 2019 (covid 2019): análisis de mortalidad hospitalaria (Fuente: elaborado por la Comisión de Vigilancia Epidemiológica de Mortalidad, sector Epidemiologia, Medicina Preventiva. Hospital Nacional Profesor Alejandro Posadas. Gentileza dra. Maria Elena Borda.)

“Nunca vi morir tanta gente en mi vida”, frase de A. que trabaja en clínica médica en donde “en general muere mucha menos gente que en terapia” cobra sentido en este marco específico (entrevistada A.). La forma de morir es “terrible”:

La situación es terrible, la enfermedad es terrible. Los pulmones se destruyen. Todas las tomografías de pulmón son iguales. Los pulmones tienen un receptor especial al que el virus se une. Eso es algo [pausa] de mandinga [pausa] algo maquiavélico. ¿Cómo puede ser que justo este virus se una específicamente al receptor pulmonar y lo destruya? Deja de existir la posibilidad de respirar. Y esto parte del contacto con el otro. Este concepto es terrible. No puedo respirar más porque me acerqué a otra persona y esa persona quizás sea la que yo más quiero. Es muy terrible… fundamentalmente el contacto con el otro que es una de las cosas que más valorábamos es lo que me produce esto que no puedo respirar más. Y si yo tengo contacto con el que más quiero puedo hacer que a él le pase esto. Este concepto es lo más terrible de todo (entrevistada C.A.).

Esta “muerte muy fea”, como la define la entrevistada C., es conocida por todo el personal hospitalario. El equipo médico y las enfermeras son las personas que más cerca se hallan de los casos de muerte y, por lo tanto, las que se enteran en primer lugar, según su ubicación en el servicio o su posición jerárquica. Sin embargo, no todos están igualmente expuestos a presenciar los fallecimientos, a ver, tocar y preparar los cadáveres. El médico confirma la muerte, pero la manipulación de los cuerpos y su preparación para ser entregado a la empresa fúnebre – como sucede en contexto de covid-19 – es un asunto del “personal de enfermería” y la administración del hospital que emite el certificado y la identificación correspondiente al cuerpo muerto que los enfermeros colocan en la bolsa que cubre y oculta al cuerpo muerto.

El manejo del cuerpo muerto, por ejemplo, el traslado de un cadáver a la camilla, la limpieza y la mortaja, son rutinas organizadas que, generalmente, se realizan por dos o más enfermeros que aprendieron sus técnicas “haciendo prácticas hospitalarias y en las morgues mientras estudiaban”. Esta formación y práctica profesional es una actividad ceremonial estructurada temporalmente, con etapas y modos de finalización que se readaptaron en el curso de la crisis pandémica. Por protocolo se acortaron los tiempos de preparación y, en ocasiones y para evitar el contagio, el cadáver conserva los instrumentos de su internación – como sondas o tubos endotraqueales – que acompañaron a la persona en vida. Al igual que para preparar un paciente para su intubación, los enfermeros manipulan los cuerpos muertos, pero son los médicos quienes anuncian a los familiares, amigos y/o cuidadores el fallecimiento. A diferencia de los enfermeros para quienes “el tratamiento de óbitos” es parte de la formación profesional, los médicos no han recibido instrucción especial ni en su lugar de trabajo ni en su etapa de formación universitaria para manipular cuerpos y para comunicar las malas noticias. Tampoco han recibido entrenamiento para desdoblarse, como lo hacen a menudo, entre sus actitudes y emociones como médico y como persona. La gestión emocional es resultado de la praxis y de involucramientos afectivos “excesivos” que, precisamente, buscarán gestionar y controlar.

En situaciones normales la comunicación se produce en una interacción cara a cara en la que el médico informa a la familia, amigos o cuidadores, de modo rápido, claro y eficaz el fallecimiento. La pandemia cambió el contexto y quitó los lenguajes gestuales y la comunicación visual, que los entrevistados añoran. Solo a mediados del 2021 se rehabilitó el contacto personal, pero entre marzo de 2020 y fines de julio de 2021 el vínculo fue exclusivamente telefónico. Así, la voz, sus tonos y timbres, la palabra “justa” y los silencios cumplieron un rol central para comunicar la muerte. Además, la noción de “morir”, como caracterización predictiva propia del personal de salud, ganó importancia y fue especialmente usada para enviar mensajes que “preparan” a los deudos para el desenlace. “Son charlas telefónicas, no ves la cara, no ves un gesto. Ir anunciando de a poco” nos relata P.D. y, prosigue, “cuando se sabe que está cada día peor, mal decir la verdad. Está mal, decirle al familiar que está mal” (entrevistado P.D.). ^26^

El derecho a saber de los familiares y/o cuidadores, y la obligación y el compromiso de decir la verdad de los médicos reconfigura la interacción en situaciones críticas como durante la covid-19. Pero no inhibieron, por supuesto, ni la preparación ni la reflexión previa sobre cómo comunicar. Ambas coexisten en las entrevistas con “lo digo como me sale” o “siempre está la cuota personal, el condimento de uno”. Y es, precisamente, entre la estandarización y la improvisación o espontaneidad que se comunican las muertes.

Nosotras, cuenta lo entrevistado J., “nos escribimos en un papel los datos del paciente y de quien atiende la llamada”. Conocer la historia clínica es parte del trabajo profesional, es tanto lo que se hace como lo que se espera y, también, lo que permite manejar con “responsabilidad” la comunicación y las interacciones que el enunciado de una muerte puede lubricar. “Informar siempre el mismo caso” genera un vínculo entre el médico y el familiar que es la base mínima para tener confianza y no dudar de la palabra que confirma y nombra la muerte. Esta declaración de gran importancia para la familia, que tiene – y nadie nos expresó haber sido desafiado cuando informa – el poder de lo incuestionable y de lo indudable, no es emocionalmente neutra. Quien recibe la noticia puede sollozar, llorar, permanecer sin hablar. Cuando estas expresiones suceden, se respetan los silencios, se escuchan los llantos como parte del acompañamiento y del control de la situación.

Por el contrario, informar la muerte de un paciente desconocido (no disponer de datos precisos de cómo aconteció el deceso) es un quiebre. “Fue muy difícil”, relata la entrevistada C., tan difícil que lo narró en dos oportunidades. Ante la pregunta:

– ¿Hay alguna situación que te haya marcado, que sea para vos un punto de quiebre?

– … [silencio]

– Me refiero a que para vos sea un antes y un después. Puede ser desde el punto de vista profesional, porque aprendiste algo puntual, un intercambio, un gesto o un rostro, o alguien que se murió.

– Claro. Claro [pausa]. Hubo una situación te diría [pausa] que había muerto durante la noche y la familia no se había enterado. Era un paciente que yo no había conocido. Un paciente que yo no llegué a conocer. No sabía cómo se había dado ese desenlace y tenía que contarles bien qué había pasado. Así que fue difícil. Hablé con la gente de guardia, leíamos la historia clínica, leí todo lo que me podía traer un poco de claridad al momento de contarles lo que había pasado y bueno contarles que había sufrido una descompensación durante la noche y bueno contarles que de esa descompensación no había podido salir y que había fallecido [pausa]. Es tremendamente distinto decir lo conocía, en algún momento había hablado con la familia, visto, ya tenías una relación podríamos decir, que tenés una especie de relación con la familia. Pero acá nada. Eso para mí fue muy difícil.

– ¿Por qué?

– En realidad, porque yo le tenía que decir a esas personas que habían perdido un familiar y yo no tenía ni conocimiento de lo que había pasado, ¿viste? No porque dude lo que se hizo o no se hizo sino porque yo había estado al margen y estaba dando posiblemente la peor noticia a esos papás. Era un chico joven, con discapacidad. Fue muy difícil, la verdad fue muy difícil, cosas lindas que nos pasaron, bueno otra cosa que fue muy fuerte para mi es que nuestros compañeros se enfermaban, que nos empezábamos a enfermar. No falleció nadie del servicio. Pero bueno con mi terapia, con la familia, con todos los recursos pudimos seguir, teníamos que seguir (entrevistada C.).

También son inconvenientes las muertes nocturnas. Debido a que quien las informa estuvo generalmente ausente cuando sucedió. Se quiebra el imaginario del acompañamiento médico al moribundo, su saber sobre el momento y la manera exacta de morir y se debe afrontar sin información propia las preguntas que la noticia dispara. Una vez más, el deudo no cuestiona la palabra médica pero sí quiere saber cómo fue el final. La distancia entre el momento que la muerte sucede a la madrugada y la notificación a los deudos que se hará en el curso de la mañana produce una nueva temporalidad, diferente a la expectativa general de la notificación rápida, casi inmediata. Hay que saber llenar en la conversación el vacío de esta espera. Es importante, para la entrevistada C., “darle pautas de que no sufrió tanto. Alivia para la familia saber que estuvo tranquila la persona, que no estaba con dolor, es muy aliviadora”. Interesarse por la existencia de algún dolor antes de fallecer es una pregunta que se espera de parte del familiar. Cuando sucede la respuesta, al parecer, siempre es no.

Cuando se le pregunta al entrevistado J. si hubo alguna muerte que recuerde especialmente, respondió con una escena particular. Conmovida – se le quiebra la voz, sus ojos se empañan y por momentos llora – elige una conversación telefónica con Natalia.

[J.] llama para decirle a un Natalia familiar que su papá había muerto, aunque su mamá continuaba internada]. No lo relacioné [a la muerte] con mi familia, con mi papá y mi mamá, sino que dije como hace una familia [silencio y con los ojos húmedos] probablemente deben estar destrozados. Yo la llamo a Natalia, la llamé ayer, le digo que su papá murió y que mejoró un montón su pulmón – el de la mamá – [silencio] esperemos que no aparezca ninguna complicación y me di cuenta de que no tendría que decirle esto, pero se lo estaba diciendo, y ella me dijo, “si doctora yo ya lo sé pero mi mamá ya pasó mucho por esto, mejora y después aparece una complicación y después aparece todo para atrás”. Yo había hablado mucho con Natalia (entrevistado J.).

La muerte comunicada a través de dispositivos que impiden la proxemia de los cuerpos y sus lenguajes no es por ello emocionalmente neutra para quien la enuncia. El teléfono como dispositivo que suple la presencia física inaugura un distanciamiento y desterritorializa la comunicación y, al mismo tiempo, acerca a los interlocutores y facilita lazos en pandemia. Cuando son varios los integrantes de un grupo familiar internados o cuando un paciente muere luego de una larga internación, la comunicación de una muerte se impregna de una “historia” de llamados telefónicos significativos y significantes que producen, en palabras del entrevistado J., que “uno pierde la distancia y ahí la muerte te afecta”. No hay desdoblamiento entre el profesional y el individuo.

## Consideraciones finales

El paso de la covid-19 en el hospital disparó un conglomerado de cambios en las prácticas y rutinas de trabajo, en la reorganización del espacio físico y, llegado el caso, desafió los modos convencionales de comunicar la muerte. Qué permanecerá y cómo lo hará es imposible todavía de precisar, aunque, como aconteció con epidemias y pandemias del pasado, después de una crisis ya no es posible una restauración, una vuelta exacta al período anterior. La pregunta: ¿de los cambios que disparó o intensificó la covid-19 qué te gustaría preservar? integró el cuestionario desde el inicio de la investigación. Cuando realizamos las entrevistas, un año y medio después del inicio de la pandemia y cuando el número de infectados y muertos comenzaba nuevamente a subir marcando la “segunda ola”, había espacio para preguntar: ¿cómo le fue al hospital con la pandemia? ¿cómo te fue a vos?

La entrevistada C., mirándome a los ojos y sin dudar, disparó: “Excelente. Nos fue excelente”. Y de inmediato y sin dejar de mirarme preguntó: “¿es ético hacerlo (la pregunta, la autoevaluación) en medio del dolor sordo que tenemos todos?”.

La pregunta abre una caja de pandora de la cual emergen diversidad de perspectivas – la experiencia no es unívoca, hay momentos significativos para los protagonistas muy diversos entre sí – y también porque no hay consenso en las ciencias sociales a partir de qué evaluar y en relación con qué: ¿la cantidad de muertes: totales y parciales? ¿de asistidos? ¿de diagnosticados? ¿desde la perspectiva de la cultura organizacional particular? ¿en relación con otros centros de salud? Las discrepancias e interrogantes aún abiertas en los estudios académicos no emergen entre los trabajadores que entrevistamos en el hospital. Nos fue “excelente”, “nos fue muy bien” fue una respuesta recurrente. ¿Con más de cien mil muertos te parece que nos fue bien? Repregunté. Somos el tercer país de Latinoamérica, continué. ^27^

– Si, pero sabes qué, hay un subregistro importantísimo de infecciones y contagios. Y también las expectativas: a partir de dónde partís y lo que imaginas que te va a suceder.

– ¿De dónde partieron? ¿Qué imaginabas?

– De un hospital fraccionado, que sufrió mucho en la gestión anterior que lo fraccionó y rompió el diálogo; que jubiló forzadamente a parte del personal y que frenaba las discusiones científicas al interior…pensábamos que íbamos a vivir una tragedia. Y no sucedió… (entrevistada M.E.). ^28^

“No saturamos” enfatiza orgulloso el entrevistado P. Yo siempre, prosigue, “tengo cuarenta personas que quieren subir a un barco, pero tengo lugar para veinte, y me las tengo que arreglar, las tengo que atender igual”. “No saturamos” afirman también seguras las entrevistadas A. y M.C.: “nadie tuvo que esperar un respirador o se murió porque no lo tuvo; no tuvimos enfermos atendidos en los pasillos y muertos apiñados en la morgue. Tuvimos todos los materiales – barbijos, trajes, camas, respiradores etc. –, nunca nos faltó nada”.

Las respuestas a la pregunta pueden entenderse a partir del concepto de *communitas* , es decir, de un intenso sentimiento de comunidad entre personas que experimentan juntas situaciones de liminalidad ( Turner, 1974 ). Este sentimiento de proximidad se entrelaza con el de colaboración y de confianza hacia y entre pares con roles y posiciones jerárquicas diferentes. “Que somos un equipo (y) que podemos confiar en el trabajo del otro” (entrevistada L.) es un futuro laboral deseable en una institución que habría gestionado con excelencia la crisis sanitaria. Además, esta forma de pensar a la institución que los propios actores expresaron en las entrevistas, con una evaluación siempre positiva en torno a lo posible, lo deseable y lo que efectivamente ocurrió (no saturar) reconstruye el paso de la pandemia por el hospital nacional Alejandro Posadas atravesado por dos elementos que también son parte del debate nacional en Argentina sobre la covid-19. El primero es lo que podríamos llamar un “espejo europeo”: la experiencia italiana, española y en menor medida la francesa de principios del 2020, con las postales de enfermos sin respiradores y muertos en los pasillos de los centros de atención, ayudó a conformar un horizonte de expectativas muy negativo y hasta catastrófico para Latinoamérica. La notoria disparidad entre los sistemas de salud de Europa y los latinoamericanos anticipaba una crisis que, finalmente, no llegó a expresarse como fuera inicialmente imaginada. Esta disparidad, precisamente, contribuyó a legitimar la autoevaluación positiva del hospital Posadas, ya que esa situación tan temida no aconteció. El segundo elemento que también ayuda a conformar esta imagen positiva es la “flexibilidad” del personal de salud y su capacidad de gestionar y alterar prácticas, espacios e itinerarios involucrados con en el tratamiento contra la covid-19. Frente a la llegada de la enfermedad, el personal de salud lo afrontó con sus saberes previos, pero al mismo tiempo desarrolló una capacidad de adaptación y exigencia muy altos que si bien no son completamente ajenos a la práctica médica en general en la situación de crisis generalizada que implicó la pandemia se incrementó de manera notable. Las terapias y sus especialistas cargaron con todo el peso de afrontar la pandemia, recortándose de otras especialidades. Ese punto también cuenta en esta autoevaluación del proceso desafiante y arduo de salvar vidas durante la covid-19.

## NOTAS

1 El primer caso de covid-19 fue notificado en la Ciudad Autónoma de Buenos Aires el 2 de marzo de 2020. El 11 de ese mismo mes la OMS decretó a la nueva enfermedad como una pandemia.

2 De las diferentes variantes del SARS-CoV-2, nuestro estudio coincide con el predominio de la variante Delta – por su tasa de contagio y por su mortalidad. La variante Ómicron, posterior de la Delta, tuvo una expansión muy rápida desde fines del 2021 y hasta mediados del mes de febrero de 2022. La expansión de esta variante, más contagiosa pero menos letal que la Delta, coincide en la Argentina con la existencia de un porcentaje importante de la población vacunada. Según los datos del Ministerio de Salud de la Nación, el 23 de febrero de 2022, el 80,22% de la población adulta residente en el país estaba vacunada.

3 Nos referimos al boletín semanal epidemiológico y estadístico covid-19, los informes de la Comisión de Vigilancia Epidemiológica de Mortalidad, así como a artículos publicados en revistas académicas resultantes de investigaciones llevadas a cabo por profesionales del hospital.

4 Los Encuentros Hospitalarios de Gestión fueron organizados por la Dirección General de Asistencia médica del Hospital A. Posadas desde mediados de 2020. Se realizan cada 15 días, de forma virtual y/o presencial, dependiendo de la situación epidemiológica, y en los cuales cada uno de los equipos del hospital (clínica médica, unidad de cuidados intensivos, epidemiologia etc.) informa sobre la situación y medidas adoptadas. Sandra Gayol fue autorizada a participar en dos de ellos referidos a mortalidad y clínica médica.

5 Las preguntas iniciales de las entrevistas fueron pensadas conjuntamente por los dos autores de este trabajo, pero las entrevistas las realizó Sandra Gayol. Ricardo González fue un actor clave para el ingreso al hospital y para alcanzar y concretar el efecto “bola de nieve” de los contactos iniciales. Nuestro especial agradecimiento.

6 Las entrevistas fueron grabadas, un solo entrevistado prefirió que no se grabara su testimonio, duraron un poco más de una hora, una de ellas se extendió por 1h45 y otra hasta 2h10. Se volvió a contactar a algunos entrevistados para desarrollar algunos puntos y/o chequear información. Con varios de ellos, también, se mantuvieron intercambios posteriores por WhatsApp. En los mismos nos compartieron artículos periodísticos sobre la pandemia porque, “te puede interesar” o reflexiones, homenajes a fallecidos o al personal de salud de Argentina o de otro país: “me acordé de vos y tal vez te sirva”. Los nombres de los entrevistados han sido modificados.

7 Los municipios de Matanza, Merlo, Moreno, Hurlinghan, Ituzaingó, Tres de Febrero y San Miguel (todos ubicados al oeste de la provincia de Buenos Aires). Disponible en: https://www.argentina.gob.ar/salud/hospital-nacional-posadas/acerca-del-hospital . Acceso en: 8 jun. 2023.

8 Entrevista presencial a L., enfermera, 46 años y 25 de servicio, realizada en el Hospital Posadas, 28 ago. 2021.

9 En los momentos de mayor contagio, entre julio y octubre de 2020, se implementó, por indicación del Ministerio de Salud de la Nación, la articulación con “hospitales de campaña” de las zonas aledañas que recibían pacientes con covid-19 que no requerían cuidados intensivos y un seguimiento especial.

10 Entrevista virtual a C.A., 60 años, médica intensivista especialista en terapia intensiva de adultos, 18 mayo 2021.

11 Entrevista presencial a L., enfermera de terapia intensiva, 28 ago. 2021. Una representación visual preparada para la televisión que muestra la demarcación al interior del “área covid” entre el “área limpia” (donde se depositan los medicamentos e instrumentos a utilizar con los pacientes) y el “área sucia” (donde se colocan los desechos) está disponible en: https://www.youtube.com/watch?v=u-cnNP0VJ2Q&t=3s . Acceso en: 2 jun. 2023.

12 Entrevista virtual a A., médica clínica, 1 jul. 2021.

13 La frase es de la médica M.E., 57 años, hace 32 que trabaja como médica y en los últimos años se especializó en epidemiología. Entrevista presencial realizada en el Hospital Posadas, 28 ago. 2021.

14 Entrevista virtual a A.P., médica clínica de 38 años, hace ocho que trabaja como médica y dos en el hospital, 5 jul. 2021. En este punto nuestras entrevistas coinciden con los resultados de la encuesta a trabajadores sanitarios realizada por la Fundación Cardiológica Argentina. La encuesta apuntó a conocer los efectos económicos, profesionales y emocionales que experimentaron dichos trabajadores y muestra que el 82% de las respuestas manifestaron haber experimentado miedo por contagiar a sus familiares y seres queridos, ansiedad e insomnio. Disponible en: https://www.comunicar-salud.com.ar/2020/08/03/44914/ . Acceso en: 3 nov. 2021. No se aclara la fecha en que se realizó la encuesta. También coincide con lo observado por Freidin et al. (2021) y Ortiz et al. (2020) .

15 Entrevista presencial a H., enfermera de 39 años, siempre trabajó en el Hospital Posadas, primero en “piso” y después en terapia intensiva, 26 jul. 2021.

16 Entrevista virtual a A.N., 34 años, quien realizó la residencia entre el 2015-2019 en el hospital. Especialista en medicina interna y desde marzo de 2020, por la pandemia, es contratada para desempeñarse en clínica médica. Entrevista realizada el 1 jul. 2021.

17 El Aspo fue anunciado por el presidente argentino el 19 de marzo de 2020. En ese momento había 128 casos de covid-19 y tres fallecidos. El primer caso se conoció oficialmente el 2 de marzo de 2020 y el “primer caso” en el hospital fue el 7 de marzo. Se trató de una mujer que estuvo internada casi dos meses en terapia intensiva y obtuvo el alta médica el 7 de mayo de 2020.

18 Entrevista presencial a M.C.A., 42 años, médica especialista en cuidados paliativos, realizada el 19 mayo 2021.

19 C., además del entrenamiento formal, cuenta y sonríe: “seguía en Instagram una enfermera de Barcelona, ‘Enfermera saturada’”.

20 Cuando pregunto a L., enfermera de terapia intensiva abocada a pacientes con covid-19, si mira para atrás, qué aprendizaje rescataría, sin dudar me dice: “aprendí, aprendimos, mucho a confiar en nuestros compañeros. Antes cada enfermero leía las indicaciones, preparaba la medicación y la suministraba. Cuando estuvimos a tope era imposible, entonces mi compañero me preparaba la medicación, yo entraba a la sala, se la daba al paciente. Antes eso era impensable. Aprendimos a confiar. Y eso está bueno”.

21 Entrevista virtual a C.P., médica de clínica médica, 6 jun. 2021.

22 El primer programa de Cuidados Paliativos en Argentina siguiendo los lineamientos de la OMS comenzó en la ciudad de Buenos Aires en 1985. En el Hospital Posadas existe una Unidad de Cuidados Paliativos de Adultos y otra Pediátrica que desde mediados de 2010 viene realizando, de manera sostenida en el tiempo, “Jornadas de Cuidados Paliativos” para difundir su existencia entre la población. En Argentina no existe aún una Ley Nacional de Cuidados Paliativos. El lanzamiento del Movimiento Hospice en Argentina fue organizado por el Hospice San Camilo en 2006.

23 Sobre el derecho a la salud como derecho humano en el contexto de pandemia por covid-19 en Latinoamérica con breves referencias al problema de morir y el final de vida; véase Pecheny (2020) . También puede consultarse las actividades implementadas por la red “Cuidados, derechos y decisiones al final de la vida” del Conicet. Disponible en: https://proyectosinv.conicet.gov.ar/riosp/ . Acceso en: 7 feb. 2023.

24 “Hay familias que no quieren ver a los pacientes porque tienen miedo a contagiarse” (entrevistado C.A.P.,). Entrevista virtual a C.A.P., médico intensivista, 67 años., realizada el 15 mayo 2021.

25 Los perfiles de los fallecidos coinciden con los conocidos a nivel nacional: comorbilidades previas, mayores de 65 años y más hombres que mujeres. La información estadística está tomada del informe de la Comisión de Vigilancia Epidemiológica de Mortalidad, sector Epidemiologia (Informe…, 2021). Agradecemos a la dra. María Elena Borda el acceso a la información y todas las aclaraciones y precisiones solicitadas en el curso de esta investigación.

26 Entrevista presencial a P.D., médico, 57 años y 32 de médico, realizada el 26 mayo 2021.

27 Al momento de realizada la entrevista la cifra oficial de muertos a nivel nacional era de 114.579 personas.

28 El sistema sanitario argentino en un contexto mayor de crisis económica y social estaba en una situación crítica que si bien es de vieja data fue profundizada en el curso de los cuatro años previos de gobierno de Juntos por el Cambio. Cuando asumió el nuevo gobierno, en diciembre de 2019, el Congreso de la Nación declaró la emergencia sanitaria, se incrementó el presupuesto en salud y el Poder Ejecutivo restableció el estatus de ministerio a la cartera de salud que había sido rebajada a secretaría.
